# Supplementary material for: Mechanisms governing the pioneering and redistribution capabilities of the non-classical pioneer PU.1
Source: Nat Commun. 2020 Jan 21;11:402. doi: 10.1038/s41467-019-13960-2 (PMC6972792; doi:10.1038/s41467-019-13960-2)
Supplement: Supplementary file 7 — Source data [file 41467_2019_13960_MOESM7_ESM.zip › Source_Data/FigureS2/FigureS2B_MotifScanOutput/KmeansCluster11/homerResults/motif11.similar.html]

motif11

## Information for motif11

T
G
C
A
C
A
G
T
A
T
C
G
G
T
C
A
C
A
T
G
A
G
C
T
T
A
G
C
T
C
G
A
A
G
C
T
  
Reverse Opposite:  

T
C
G
A
A
G
C
T
A
T
C
G
T
C
G
A
G
T
A
C
C
A
G
T
T
A
G
C
G
T
C
A
A
C
G
T
  

|  |  |
| --- | --- |
| p-value: | 1e-42 |
| log p-value: | -9.750e+01 |
| Information Content per bp: | 1.645 |
| Number of Target Sequences with motif | 307.0 |
| Percentage of Target Sequences with motif | 9.45% |
| Number of Background Sequences with motif | 1699.7 |
| Percentage of Background Sequences with motif | 3.76% |
| Average Position of motif in Targets | 102.5 +/- 55.4bp |
| Average Position of motif in Background | 100.0 +/- 61.6bp |
| Strand Bias (log2 ratio + to - strand density) | 0.1 |
| Multiplicity (# of sites on avg that occur together) | 1.04 |
| Motif File: | file (matrix) reverse opposite |

### Similar de novo motifs found

|  |  |  |  |  |  |  |  |
| --- | --- | --- | --- | --- | --- | --- | --- |
| Rank | Match Score | Redundant Motif | P-value | log P-value | % of Targets | % of Background | Motif file |
| 1 | 0.908 | T G C A C G A T A C T G C T G A T A G C C G A T A T G C G T C A A C G T A G T C G A C T A C T G | 1e-38 | -88.221007 | 6.89% | 2.39% | motif file (matrix) |
| 2 | 0.908 | T C G A C A G T A T C G C T G A A T C G C A G T G T A C G C T A A C G T A G T C G C A T | 1e-37 | -85.510980 | 5.88% | 1.86% | motif file (matrix) |
| 3 | 0.950 | T G C A A C G T A C T G C G T A A T G C A C G T A G T C G T C A | 1e-35 | -81.492707 | 8.62% | 3.59% | motif file (matrix) |
| 4 | 0.950 | A C G T A C T G C G T A A C T G A C G T A G T C C G T A | 1e-31 | -72.962745 | 9.63% | 4.49% | motif file (matrix) |
| 5 | 0.899 | T A C G G T C A A C G T A C T G C G T A A T C G A C G T A G T C G T C A A G C T A G T C G A C T G A C T | 1e-26 | -60.042271 | 2.22% | 0.39% | motif file (matrix) |
| 6 | 0.941 | T A G C G T C A C G A T A T C G T C G A A T G C A C G T A T G C G T C A C A G T | 1e-25 | -59.855694 | 3.05% | 0.76% | motif file (matrix) |
| 7 | 0.828 | G A C T A C T G G T C A T A C G A C G T T A G C T G C A A T G C A G C T A G T C | 1e-23 | -55.045235 | 2.37% | 0.50% | motif file (matrix) |
